# Narrowing the focus on the assessment of psychosis-related PTSD: a methodologically orientated systematic review

**DOI:** 10.3402/ejpt.v7.32095

**Published:** 2016-09-27

**Authors:** Miriam Fornells-Ambrojo, Alison Gracie, Chris R. Brewin, Amy Hardy

**Affiliations:** 1 Research Department of Clinical, Educational and Health Psychology, University College London, London, UK; 2 Department of Psychology, Institute of Psychiatry, Psychology and Neuroscience, King's College London, London, UK

**Keywords:** Psychosis, trauma, PTSD, psychosis-related PTSD, assessment

## Abstract

**Background:**

Posttraumatic stress disorder (PTSD) in response to psychosis and associated experiences (psychosis-related PTSD, or PR-PTSD) is the subject of a growing field of research. However, a wide range of PR-PTSD prevalence rates has been reported. This may be due to definitional and methodological inconsistencies in the assessment of PR-PTSD.

**Objective:**

The focus of the review is two-fold. (1) To identify factors that enhance, or detract from, the robustness of PR-PTSD assessment and (2) to critically evaluate the evidence in relation to these identified criteria, including the impact on PR-PTSD prevalence rates.

**Method:**

Four quality criteria, whose development was informed by mainstream PTSD research, were selected to evaluate findings on PR-PTSD prevalence. Two criteria related to assessment of psychosis-related stressors (participant identification of worst moments of discrete threat events; psychometrically robust trauma measure) and two focussed on PR-PTSD symptom measurement (adequate time elapsed since trauma; use of validated PTSD interview) in the context of psychosis.

**Results:**

Twenty-one studies of PR-PTSD, with prevalence rates ranging from 11 to 51%, were evaluated. Fourteen studies (67%) used robust PTSD measures but PR-trauma was not specifically defined or assessed with validated measures. Eleven studies (52%) assessed PTSD before sufficient time had elapsed since the trauma. Due to significant methodological limitations, it was not possible to review PR-PTSD rates and provide a revised estimate of prevalence.

**Conclusions:**

Methodological limitations are common in existing studies of PR-PTSD prevalence. Specific recommendations for improving assessment of psychosis-related trauma are made to guide the development of this new and emerging field. The review concludes with a proposed conceptualisation of PR-PTSD in the context of current diagnostic systems. The utility of the PR-PTSD term and its theoretical underpinnings are discussed.

**Highlights of the article:**

- Overall, robust measures of PTSD were employed but psychosis-related trauma was not specifically defined and time since trauma was not consistently recorded
- Given the above methodological limitations in the assessment of PR-PTSD, a revised prevalence rate is not provided
- A new conceptualisation of PR-PTSD in relation to the type of stressor is proposed in the context of current diagnostic systems

Posttraumatic stress disorder (PTSD) is precipitated by traumatic events that meet specific objective criteria in the Diagnostic and Statistical Manual of Mental Disorders, 5th ed. (*DSM-5*; American Psychiatric Association, 2013) but are more broadly defined as being exceptionally threatening or catastrophic in the proposed 11th edition of the International Statistical Classification of Diseases and Related Health Problems (*ICD-11*: Maercker et al., 2013). Rates of trauma and PTSD are high in people with psychosis compared to the general population (Achim et al., 2011; Grubaugh, Zinzow, Paul, Egede, & Frueh, 2011). Shaner and Eth's (1989) proposal that the experience of psychosis *itself* can be traumatic and lead to PTSD has received increased attention in recent years. Berry, Ford, Jellicoe-Jones, and Haddock (2013) termed this construct “psychosis-related PTSD” (PR-PTSD), defining it as “PTSD induced as a result of the experience of psychosis and upsetting or potentially traumatic treatment experiences.” Research has grown in recent years but to date there has been little consideration of the conceptualisation and assessment of PR-PTSD, which we argue is necessary to inform theory and practice developments in the area.

PR-PTSD occurs in relation to distressing psychotic symptoms (e.g., hallucinations) or associated experiences (e.g., coercive treatments). For example, James was detained under the Mental Health Act 6 months ago when he had a persecutory belief that his family wanted to kill him. His psychotic symptoms had resolved and he no longer believed his family intended to harm him. However, he frequently had intrusive, vivid images of incidents when he believed his family were about to attack him. James avoided any reminders of what had happened, and felt on guard and anxious about the problems returning.

Comorbid PTSD in people with psychosis can lead to worse outcomes (Achim et al., 2011). Therefore, it is important to understand and treat PR-PTSD. Indeed, because it is precipitated by psychosis itself, PR-PTSD may cause specific difficulties in recovery (Gumley & Schwannauer, 2006). However, evaluation of the occurrence, correlates, and treatment of PR-PTSD is hindered by the wide range in reported PR-PTSD prevalence rates (e.g., 11–67%; Berry et al., 2013). We propose this substantial variation may be attributable to differences in PR-PTSD definition and assessment, which are understandably inherent in a developing field.

The current review therefore examines the reliability and validity of research on psychosis-related trauma, and of PR-PTSD assessment, by evaluating published studies against a priori gold standard diagnostic criteria for PTSD in the context of psychosis. It builds on Berry et al.'s (2013) review of prevalence rates of PR-PTSD by specifically aiming to clarify the reasons for the variation in PR-PTSD prevalence.

There has been extensive debate over the definition of the traumatic stressor in PTSD diagnosis (Brewin, Lanius, Novac, Schnyder, & Galea, 2009) which is particularly pertinent to psychosis. Specifically, in relation to internally generated, *subjectively* threatening events (such as hallucinations), neuroscientific evidence attests they may be supported by similar neural processes to actual memories (Schacter, Addis, & Buckner, 2007) and *experienced* as objective events (e.g., Allen, Larøi, McGuire, & Aleman, 2008). Furthermore, case histories and research studies testify to the high levels of threat created by psychotic symptoms and report traumatic reactions including re-experiencing (Gumley & Schwannauer, 2006; Shaw, McFarlane, Bookless, & Air, 2002).

There are challenges to identifying an individual's worst psychosis-related traumatic event, arguably because, as in PTSD due to physical illness (Kangas, Henry, & Bryant, 2002) or prolonged sexual or physical abuse (Roth, Newman, Pelcovitz, Van der Kolk, & Mandel, 1997), the experience of psychosis may be chronic and/or repeated with individual traumatic events occurring over an extended period. Nonetheless, evidence suggests subjective threats associated with psychosis can be traumatic, leading to intrusive memories that are threatening in the “here and now” and result in PR-PTSD.

## Aims

The central aims of the review were to (1) identify factors that enhance, or detract from, the robustness of PR-PTSD assessment and (2) evaluate the evidence in relation to these identified criteria, including their impact on PR-PTSD prevalence rates.

## Method

### Criteria for robust PR-PTSD assessment

Identifying factors essential to reliable and valid PR-PTSD diagnostic assessment, for the purpose of selecting criteria to evaluate PR-PTSD studies, was achieved in two stages. First, the second author (AG) consulted the PTSD literature to identify key methodological factors in mainstream PTSD assessment. Second, criteria were discussed by the authors, and consensus was reached on essential criteria for reliable and valid PR-PTSD diagnostic assessment. The following factors were selected for their consistency with the research evidence base, best practice clinical guidelines, and recently revised *DSM-5* and proposed *ICD-11* (Maercker et al., 2013) diagnostic criteria for PTSD. There are four factors, two related to assessment of psychosis-related trauma, and two to PR-PTSD measurement. They are described below and summarised in Table 1 alongside their hypothesised impact on prevalence rates.

**Table 1:** Rating system for evaluating quality of PR-PTSD prevalence studies

Rating criteria		Impact on prevalence rate
I. Assessment of Psychosis-related traumatic stressors		
1 Definition of psychosis-related trauma	0=not defined/unclear if trauma is psychosis-related 1=pre-defined as either a specific aspect of psychosis (e.g., involuntary admission) or a specific episode (e.g., combined experience of symptoms and hospitalisation at last episode) 2=self-identified “worst moment” of psychosis-related trauma	Risk of over/under estimation
2 Measurement of psychosis-related trauma	0=no measure used or procedure not reported 1=unvalidated measure 2=validated measure	Risk of over/under estimation
II. Assessment of PR-PTSD		
3 Time since trauma	0=less than a month/not reported 1=possibly less than a month 2=at least 1 month after the specific event	Risk of over-estimation: Higher quality rating associated with lower prevalence
4 PR-PTSD assessment tool	N/A=prevalence not reported 0=self-report 1=validated interview, including CAPS 2=CAPS for Schizophrenia (CAPS-S) or equivalent	Risk of over/under estimation

#### Definition of the psychosis-related trauma

The definition of “psychosis-related trauma” was limited to highly threatening, specific events, thereby minimising the diagnosis of PR-PTSD where another diagnosis, such as “post-psychotic depression” may be more appropriate. Clearly defining past, *specific* event(s) is important to assist the distinction between traumatic *memories* of psychosis-related events and ongoing, current experiences associated with psychosis, in order to minimise overestimation of PR-PTSD. Conversely, if the traumatic stressor is instead defined a priori by the researcher (e.g., hospitalisation only events), the respondent might report reactions to event(s) other than their worst psychosis-related traumatic experience, leading to underestimation of PR-PTSD. Therefore, studies received a higher quality rating if participants selected their most distressing psychosis-related event, as in mainstream PTSD research (Green, 1996). Lower ratings could be associated with over/under estimation or PR-PTSD.

#### Measurement of psychosis-related trauma

In mainstream PTSD research, validated measures (e.g., Trauma History Questionnaire; Green, 1996) enhance the accuracy of trauma measurement. Carlson et al. (2011) propose that psychometric properties to be assessed in trauma measures include *content validity*, by the specification of target domains and dimensions; *convergent validit*y, by examining reported rates in different populations (e.g., comparing patients with psychosis who have been coercively admitted to hospital with patients with psychosis who have never been admitted); *criterion-validity* by comparing data gathered in the PR-trauma measure with information provided in medical records or detailed information elicited during interview assessment of psychosis; *test*–*retest reliability* and *inter-rater reliability* when applicable. The highest rating was given to studies using psychosis-related trauma measures with reported psychometric properties. Depending on the sensitivity and specificity of non-validated measures, a lower quality rating might be associated with over or under estimation PR-PTSD.

#### Time since trauma when assessing PR-PTSD

Acute stress symptoms are *common* in the immediate aftermath of trauma, but for many diminish over time. Consequently, diagnostic frameworks stipulate PTSD symptoms must have persisted for several weeks (proposed *ICD-11*; Maercker et al., 2013), or 1 month (*DSM-5*), after a traumatic event for a diagnosis to be given. Therefore, studies ensuring at least a month between the psychosis-related stressor and PR-PTSD assessment received the highest rating, as not allowing sufficient time delay might be associated with an overestimation of prevalence.

#### Method of PR-PTSD assessment

Validated PTSD interviews are more reliable than self-report measures (McDonald & Calhoun, 2010) and in mainstream PTSD research the Clinician-Administered PTSD Scale (CAPS; Blake et al., 1995) is widely accepted as the gold standard (Kang, Natelson, Mahan, Lee, & Murphy, 2003). In PR-PTSD research, assessment is complicated by the phenomenological overlap between symptoms of psychosis and the key symptoms of PTSD. This means there is a risk that distress and impairment due to *current* symptoms of psychosis and related experiences may be measured rather than due to *past* memories of psychosis (Tarrier, 2005). Semi-structured interviews allow the careful differentiation of PTSD symptoms from those due to co-morbid disorders (McDonald & Calhoun, 2010). Therefore, studies were rated as having adequate quality if they used validated interviews, such as the CAPS (Blake et al., 1995) and were given the highest quality rating if they used the CAPS for Schizophrenia (CAPS-S; Gearon, Bellack, & Tenhula, 2004) to assess PR-PTSD. This is because the CAPS-S includes additional prompts to ensure post-traumatic symptoms are discriminated from psychosis. The impact of PR-PTSD assessment quality ratings on prevalence would depend on the sensitivity and specificity of the measures.

### Evaluating evidence in relation to identified criteria

#### Procedure

**Fig. 1:**
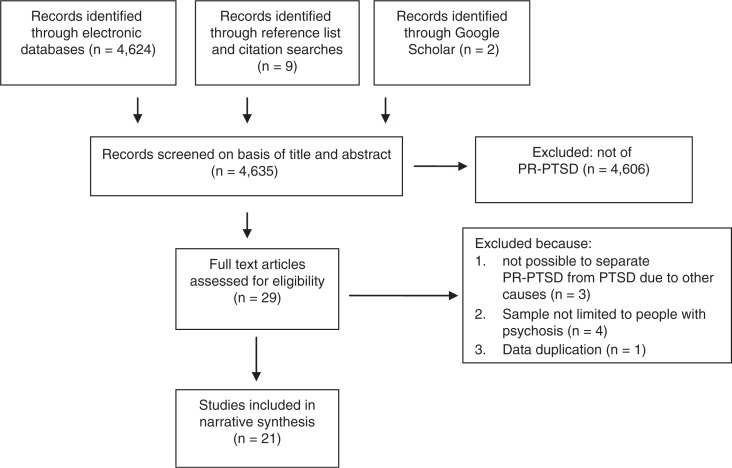
Selection of studies.

EMBASE, Medline, PsycINFO, and Published International Literature on Traumatic Stress (PILOTS) databases were searched from 1990 to 2016, week 12 using the following search terms: (post-traumatic OR posttraumatic OR ptsd OR trauma) AND (psychosis OR schizophrenia OR psychotic). Reference lists, citations and Google Scholar were also searched. Studies were included if they were of PR-PTSD; used a standardised measure to assess PR-PTSD; were quantitative; and were published in English, in a peer-reviewed journal. The final sample included 21 studies (Fig. 1).

Quality ratings of studies were carried out by the second author (AG). A subsample of the studies (*n=*9) was also rated “blind” by authors AH and MFA. Inter-rater reliability analyses for each of the four quality factors described in Table 1 yielded Kappas from 0.80 (*p*=0.010) to 1.00 (*p*<0.0001), indicating substantial, to almost perfect, agreement (Landis & Koch, 1977).

## Results

Sixteen studies were retrospective and the remaining three longitudinal (Brunet, Birchwood, Upthegrove, Michail, & Ross, 2012; McGorry et al., 1991; Meyer, Taiminen, Vuori, Aijala, & Helenius, 1999). Two studies (Priebe, Bröker, & Gunkel, 1998; Tarrier, Khan, Cater, & Picken, 2007) focused on trauma and PTSD linked to treatment experiences alone, while the remainder investigated reactions to psychotic symptoms as well. PR-PTSD prevalence ranged from 11 to 51% (Mean=32.7%, Median 31.0%) (Table 2).

**Table 2:** Summary of studies included in narrative synthesis of quality of PR-PTSD prevalence assessment

	Country	N	Age		FEP	% Non-affective	Psychosis assessment	Trauma assessment					PR-PTSD assessment			PR-PTSD Prev. %
	
								PR-trauma definition	Trauma type			PR trauma measure	Diag. assess	Time		
		
			M	SD					P %	T %	C %			Month	From	
Priebe et al. (1998)	GER	105	38.6	9.4	N	100	BPRS PSE	Experience of		✓ 100		Study's own questions	PTSD I.	41.4 (40.7)	D	51
Shaw et al. (1997)	AUS	45	29.8	10.9	N	71	CIDI FCRS	Experience of			✓ 93	CIDI Study's own questionnaire: HEQ	CAPS	0	D	49
Bendall et al. (2012)	AUS	36	21.4	3.4	Y	67	PANSS	Experience of			✓	–	IES-R	9.8 (7.33)	T	47
McGorry et al. (1991)	USA	36	25.0	4.8	Y	67	SANS	Experience of			✓	–	PTSD S.	4 11	D	46 35
Abdelghaffar et el. (2016)	Tunisia	52	27.6	5.6	Y	73.1	PANSS	Experience of	✓	✓		Study's own question	CAPS	0–12	D	42.3
Tarrier et al. (2007)	UK	35	24.9	6.3	Y	NR	PANSS	Experience of		✓ 80		Study's own questions	CAPS-S	0	D	38
White and Gumley (2009)	UK	27	38.9	10.3	N	100	PANSS	Worst moment			✓	Study's own questions	CAPS-S	72.3 (56.3)	D	37
Jackson et al. (2004)	UK	35	25.8	5.1	Y	100	KGV	FEP		✓ 77	✓	HEQ	PTSD S.	18	A	31
Lu et al. (2011)	USA	50	36.8	11.4	N	70	BPRS	Worst moment	✓ 45	✓ 78		PATS	CAPS/PDS	>1.0	T/S	28
Paksarian et al. (2014)	USA	395	27	21–34	N	74	BPRS	Worst moment		✓ 69		Study's own question	–	120	D	26
Mueser et al. (2010)	USA	38	22.5	–	Y	66	BPRS	Worst moment	✓ 68	✓ 53		PATS	CAPS/PDS	>1.0	T/S	24
Centofanti et al. (2005)	AUS	20	33.4	5.6	N	95	BPRS	Experience of			✓	HEQ	CAPS	7.75 (3.4)	D	25
Berry et al. (2015)	UK	50	37.7	100	N	100	PANSS	Most distressing	✓ 82	✓ 98		PEQ	IES-R	>1.0	T	24^P^ 18^T^ 30^C^
Kennedy et al. (2002)	USA	30 50	35.2	11.9	N	100 60	–	–	✓ 60			Study's own (trauma categories)	Penn I.	–	–	23 40

	Country	N	Age		FEP	% Non-affective	Assess. Psychosis	Trauma assessment					PR-PTSD assessment			PR-PTSD Prev. %
	
								PR-trauma definition	Coverage			PR trauma measure	Diag. assess	Time point		
		
			M	SD/IQR					P %	T %	C %			Month	From	

Sin et al. (2010)	SIN	61	25.8	6.6	Y	93.4	PANSS	Experience of	✓	✓		–	CAPS	3.8	T	20
Brunet et al. (2012)	UK	39	22.4	–	Y	94	DoT BAVQ VTS	Intrusion	✓ 28	✓ 38		Presence of distressing intrusive memories about past events	PSS-I	18	A	18
Turner et al. (2013)	UK	50	24.5	–	Y	100	–	Worst moment			✓	Study's own question	IES-R	>1.0	D	14
Meyer et al. (1999)	FIN	46	40.8	12.1	N	100	PANSS	Experience of	✓	✓		–	CAPS	0	D	11
Harrison and Fowler (2004)	UK	38	36.5	11.1	N	100	PANSS	Experience of	✓	✓		–	–	48	D	–
Chisholm et al. (2006)	UK	36	34.1	15.0	N	100	BPRS	Most difficult period of episode			✓	–	–	4.8 (3.6)	D	–
Beattie et al. (2009)	UK	44	37.5	11.5	N	91.5	KGV	Most distressing	✓ 62	✓		AES	–	1.1 (2.0)	D	–

“–”: Not specified; FEP: first episode psychosis. *Country*: UK: United Kingdom; AUS: Australia; Sin: Singapore; USA: United States of America; Fin: Finland; GER: Germany; Diagnosis =% non-affective psychosis. *Assessment of psychosis*: FCRS: Factor Construct Rating Scales; CIDI: Composite International Diagnostic Interview; DoT: Details of Threat Questionnaire; BAVQ-R: Beliefs About Voices Questionnaire-Revised; VTS: Voice Topography Scale; *Trauma assessment*: P: psychotic symptoms related trauma; T: treatment experiences related trauma; C: combined psychosis and treatment experiences related trauma; *PR-Trauma measure*: HES: Hospital Experiences Questionnaire; PATS: PTSD Assessment Tool for Schizophrenia; PEQ: Psychiatric Experiences Questionnaire; AES: Admission Experience Survey; *PR-PTSD assessment*: CAPS: Clinician Administered PTSD Scale; CAPS-S: Clinician-Administered PTSD Scale for Schizophrenia; PDS: The Posttraumatic Diagnostic Scale; IES-R: Impact of Events Scale-Revisited; PTSD S: PTSD scale *Time of PR-PTSD assessment* **:** no. of months, or mean months (SD) since discharge (D) acute episode (A) or time in treatment (T).

### Evaluation of studies in relation to the PR-PTSD assessment criteria

Ratings for the four quality assessment factors are summarised in Table 3. No study achieved top quality ratings for all four criteria. Only a small minority of studies reached the maximum score (i.e., “2”) for the trauma definition (I.1) and the assessment of PR-PTSD (II.3 and 4) criteria. No study received the maximum score in the measurement of trauma (I.2).

**Table 3:** Quality factors related to assessment of psychosis-related trauma and PR-PTSD in prevalence studies of PR-PTSD

Study and country	I. Assessment of PR-trauma		II. Assessment of PR-PTSD		Overall rating (averaged)
	
	1. Trauma definition	2. Trauma measure	3. Time since trauma	4. PR-PTSD assessment	
Lu et al. (2011)	2	1	2	1	1.5
Mueser et al. (2010)	2	1	2	1	1.5
White and Gumley (2009)	2	0	0	2	1
Tarrier et al. (2007)	1	1	0	2	1
Centofanti et al. (2005)	1	1	1	1	1
Priebe et al. (1998)	1	1	1	1	1
Paksarian et al. (2014)	2	0	1	0	0.75
Jackson et al. (2004)	1	0	1	1	0.75
Shaw et al. (2002)	1	1	0	1	0.75
Brunet et al. (2012)	1	0	1	1	0.75
Berry et al. (2015)	2	1	0	0	0.75
Chisholm et al. (2006)	1	0	1	N/A	0.67
Beattie et al. (2009)	2	0	0	N/A	0.67
Turner et al. (2013)	2	0	1	0	0.60
Meyer et al. (1999)	1	0	0	1	0.50
McGorry et al. (1991)	1	0	1	0	0.50
Abdelghaffar et el. (2016)	1	0	0	1	0.5
Harrison and Fowler (2004)	1	0	0	N/A	0.33
Bendall et al. (2012)	1	0	0	0	0.25
Sin et al. (2010)	0	0	0	1	0.25
Kennedy et al. (2002)	0	0	0	0	0

Each criterion could be rated 0–2, with higher ratings indicating higher quality.

#### Psychosis related trauma assessment

Only seven studies (Beattie, Shannon, Kavanagh, & Mulholland, 2009; Berry, Ford, Jellicoe-Jones, & Haddock, 2015; Harrison & Fowler, 2004; Lu et al., 2011; Mueser, Lu, Rosenberg, & Wolfe, 2010; Paksarian et al., 2014; Turner, Bernard, Birchwood, Jackson, & Jones, 2013; White & Gumley, 2009) defined trauma as participants’ self-generated worst moment of psychosis. Other studies used alternative definitions such as treatment experiences *alone* (Priebe et al., 1998; Tarrier et al., 2007); symptoms *alone* (Kennedy et al., 2002); or symptoms and treatment experiences assessed separately (Berry et al., 2015; Harrison & Fowler, 2014; Lu et al., 2011; Meyer et al., 1999; Mueser et al., 2010); and the *overall* experience of the last episode (Bendall, Alvarez-Jimenez, Hulbert, McGorry, & Jackson, 2012; Centofanti, Smith, & Altieri, 2005; Chisholm, Freeman, & Cooke, 2006; Jackson, Knott, Skeate, & Birchwood, 2004; McGorry et al., 1991; Shaw, McFarlane, & Bookless, 1997). In the remaining study (Brunet et al., 2012), participants were asked about *any* intrusive memories, and those related to psychosis extracted, risking under-reporting PR-PTSD prevalence as only one event was recorded per participant.

There were no available instruments assessing psychosis-related trauma with reported validity and reliability in the reviewed studies. Therefore, no studies met the highest score for this criterion, and only two studies (Lu et al., 2011; Mueser et al., 2010) used a semi-structured interview, the PTSD Assessment Tool for Schizophrenia (PATS; Williams-Keeler, 1999), assessing traumatic memories of *both* symptoms and treatment. The Hospital Experiences Questionnaire (HEQ), covering treatment experiences and its consequences at any point in time (including current experiences) was developed by Shaw et al. (1997) and was used in two further studies (Centofanti et al., 2005; Jackson et al., 2004). Berry et al., (2015) used the Psychiatric Experiences Questionnaire (PEQ; Cusack, Freuh, Hieres, Suffoletta-Maieries, & Bennett, 2003), also exclusively measuring adverse hospital events and the two remaining studies only assessed treatment experiences using their own semi-structured interviews (Priebe et al., 1999; Tarrier et al., 2007). Assessment of psychotic symptoms as possible traumatic events was generally less explicitly described. Shaw et al. (1997) captured the “psychosis” trauma component by assessing distress and intrusions in relation to psychotic symptoms elicited by the Composite International Diagnostic Interview (CIDI; WHO, 1993). Remaining studies did not use a trauma measure.

#### PR-PTSD assessment

PTSD may be diagnosed only several weeks to a month after the end of the traumatic stressor. This quality factor was difficult to rate as the trauma was often not a participant's self-generated discrete event or period of events with a defined beginning and end, and information about the termination of broader events (e.g., symptom remission or time since discharge) was unclear. Only two studies^1^ (Lu et al., 2011; Mueser et al., 2010) stated overtly that the criterion had been met. Of the remaining studies, in six an inadequate length of time had elapsed (Beattie et al., 2009; Berry et al., 2015; Harrison & Fowler, 2004; Meyer et al., 1999; Shaw et al., 1997; Tarrier et al., 2007) as some participants were assessed as inpatients or just after discharge, where trauma was defined as hospitalisation. In a further five necessary information was not provided (Abdelghaffar, Ouali, Jomli, Zgueb, & Nacef, 2016; Bendall et al., 2012; Kennedy et al., 2002; Sin et al., 2010; White & Gumley, 2009). The remaining studies ensured adequate time since *one* but not all aspects of trauma (Brunet et al., 2012; Centofanti et al., 2005; Chisholm et al., 2006; Jackson et al., 2004; McGorry et al., 1991; Paksarian et al., 2014; Priebe et al., 1998; Turner et al., 2013).

Two studies used the CAPS-S (Tarrier et al., 2007; White & Gumley, 2009) and obtained the highest rating in relation to PR-PTSD assessment criterion. A further 10 studies used the CAPS (Abdelghaffar, et al., 2016; Centofanti et al., 2005; Jackson et al., 2004; Meyer et al., 1999; Shaw et al., 1997; Sin et al., 2010) or another validated interview (Brunet et al., 2012; Lu et al., 2011; Mueser et al., 2010; Priebe et al., 1998). The remaining five studies used a self-report measure (Bendall et al., 2012; Berry et al., 2015; Kennedy et al., 2002; McGorry et al., 1991; Paksarian et al., 2014; Turner et al., 2013).

### Impact of quality on reported PR-PTSD prevalence rates

PR-PTSD prevalence rates amongst four of seven studies that achieved the maximum quality score (i.e., “2”) in any of the four criteria ranged between 24 and 30% (Berry et al., 2015; Lu et al., 2011; Mueser et al., 2010; Paksarian et al., 2014). Of the three remaining studies, White and Gumley (2009) reported a higher prevalence of 37%, which could be attributed to selection bias, as they specifically recruited participants who were experiencing “ongoing distress associated with memories of … psychosis” (p. 842). Moreover, this study and Tarrier et al. (2007), also reporting PR-PTSD rates of 38%, failed to comply with the time since trauma criterion (i.e., scored “0”), which could result in over-estimation. Lastly, Turner et al. (2013), with a higher rating on the time since trauma criterion (i.e., “1”), identified a relatively low prevalence rate of 11%. It is possible that this is accurate, but the absence of a validated PR-trauma measure and use instead of a question about “most distressing or traumatic experience … in relation to mental illness or nervous breakdown” (p. 169) could have resulted in under identification of PR-trauma. Increased sensitivity could have been achieved with a validated PR-trauma measure such as the PATS (Williams-Keeler, 1999), as it covers a range of PR-trauma experiences (e.g., Did you hear voices that threatened to hurt you…?). However, it is not possible to conclude that a revised PR-PTSD prevalence rate is likely to fall between 24 and 30% simply because of the apparent convergence between the four studies with top individual ratings, as each of them received a lower rating for at least two other criteria.

In sum, overall quality of studies was fairly low when compared against the criteria. Studies reporting PR-PTSD prevalence mostly used a validated interview measure of posttraumatic stress. However, the majority of studies employed a poor definition and assessment of psychosis-related trauma, and lacked clarity regarding the time elapsed prior to PR-PTSD assessment. As no study achieved the highest rating across all four factors, it was not possible to calculate a revised PR-PTSD prevalence rate.

## Discussion

This review aimed to clarify the reasons for the wide range of reported PR-PTSD prevalence rates by evaluating psychosis-related trauma and PR-PTSD assessment against gold standard criteria. Although overall studies used robust PTSD measures, PR-trauma was often not specifically defined or assessed with validated measures, contributing to a lack of information regarding time elapsed since trauma. A revised PR-PTSD prevalence was not calculated due to reported methodological weaknesses of studies.

The prevalence of PTSD in psychosis has been estimated to be around 16% (95% CI, 4.0–21%) (Achim et al., 2011; De Bont et al., 2015). In conjunction with our findings of inadequate methodological rigour, this suggests that addressing assessment inconsistencies will lead to a substantial revision in prevalence rates. We discuss the methodological issues below, and outline a possible conceptualisation of PR-PTSD.

### Assessment recommendations

Psychosis-related trauma assessment emerged as one of the weakest aspects of the studies. For the most part, studies used diverse and inconsistent definitions of psychosis-related trauma, and unvalidated assessment tools, which partly reflect the uncertain status of PR-PTSD in relation to *DSM-5* PTSD. Existing measures reviewed here, such as the PATS (Williams-Keeler, 1999) may be a useful starting point, but we recommend further work is carried out to develop a reliable and valid measure of psychosis-related trauma (Carlson et al., 2011).

Associations between positive and PTSD symptoms are commonly reported in both psychosis and PTSD samples (Grubaugh et al., 2011). Psychotic symptoms can contribute to a sense of current threat (Gumley & Schwannauer, 2006) and may in themselves constitute ongoing trauma (Bendall et al., 2012). Current threat related to *past* events can be separated from that due to current concerns by orienting participants to maintain a focus on memories, anchoring questions to the traumatic stressor (Chisholm et al., 2006; Harrison & Fowler, 2004). Although a relatively new measure, the CAPS-S (Gearon et al., 2004) is recommended for future research as it provides in-depth assessment, and specific prompts, to support the differentiation of PR-PTSD symptoms from those of current psychosis and other comorbidities. This includes assessing the temporal relationship between the occurrence of trauma and the onset of symptoms, and the respondent's appraisal of the relationship between reported events and possible post-traumatic symptomatology. As in the general PTSD literature, and given the high prevalence of post-psychotic depression (Iqbal, Birchwood, Hemsley, Jackson, & Morris, 2004), we recommend that assessors examine the congruence between the content of the trauma and reported intrusions, and ensure that intrusions are involuntary, vivid, sensory-perceptual intrusions with a sense of occurring in the “here and now,” in order to distinguish them from more contextualised intrusive autobiographical memories that are found in psychosis and other disorders (Brewin, Gregory, Lipton, & Burgess, 2010). Further research will also need to understand the interplay between other co-morbid presentations in psychosis, such as anxiety and substance misuse, as well as contextual factors such as prior trauma and coping (Simpson & Miller, 2002; Van Nierop et al., 2015).

#### Nosological considerations

Existing studies examining PR-PTSD are relatively scarce and the current review has highlighted methodological quality concerns, particularly the absence of a uniform definition. The term PR-PTSD has been used thematically, covering traumatic stressors of both treatment and psychotic experiences, to capture the overall challenges faced by people with psychosis that might give rise to PTSD. We support the continued use of the term “psychosis-related PTSD” for its clinical and research utility, highlighting the importance of screening for post-traumatic reactions associated with the illness itself and its treatment.

We summarise next how to conceptualise psychosis as a stressor leading to PTSD in the context of current diagnostic systems. In line with earlier recommendations that the PTSD diagnosis should focus on core PTSD phenomena and reduce emphasis on trauma criteria (Brewin et al., 2009), we also consider theoretical implications for future research on psychosis-related trauma and PTSD.

First, individuals with psychosis could be concurrently diagnosed with PTSD in respect of objectively traumatic events unrelated to psychosis (such as sexual or physical assault) if they meet DSM-5 Criterion A (Table 4 (i)). Psychotic symptoms may predate or emerge after PTSD onset.

**Table 4:** A conceptualisation of post-traumatic stress reactions in psychosis and recommendations for adaptations to assessment

Traumatic stressor	Psychosis-related trauma	Distorted reality-trauma	ICD-10/11 stressor (no formal criterion)	DSM-5 criterion A	Adaptations to assessment?	

					Trauma	PTSD
(i) Objectively threatening event involving threatened death, actual or threatened serious injury, or actual or threatened sexual violence (e.g., traffic accident, rape)			✓	✓	No	Yes^b^
(ii) Objectively threatening event associated with experience of psychosis (e.g., forced restraint in hospital).	✓		✓	✓	No	Yes^b^
(iii) Primary anomalous experience (e.g., voice saying “I'm going to kill you” or vision of attacker with a knife).	✓	✓	✓		Yes^a^	Yes^b^
(iv) Delusional appraisals of experience (e.g., car approaching is interpreted as a sign persecutors are coming to kill the person).	✓	✓	✓		Yes^a^	Yes^b^

An individual could of course experience an objectively threatening event (traumatic stressor types i, ii) while also suffering from on-going threatening psychotic experiences (traumatic stressor types iii, iv). We propose that categories (iii) and (iv) be reserved for instances of traumatic stressors that are threatening unusual experiences in the absence of an objectively threatening event (i, ii). *Assessment recommendations*:

a Detailed assessment of subjective trauma including identification and description of discrete event(s) to aid discrimination from current psychotic symptomatology and assess whether sufficient time lapse of 1 month since the index PR-trauma event

b careful anchoring of PTSD symptoms to historic objective or subjective trauma and *not current subjective psychotic threat* to ensure PR-PTSD symptoms are associated with past events.

Second, when PTSD arises from objectively threatening events related to treatment or associated with psychosis (Table 4 (ii)), DSM-5 Criterion A would also be met. As mentioned above, in this case the label “psychosis-related PTSD” has been applied to thematically group investigations looking at the impact of psychosis and to assist clinical practice.

Third, PTSD symptoms can arise in relation to experiences of distorted reality that are traumatic such as symptoms of psychosis (Table 4 (iii, iv). We propose that *distorted reality PTSD* should be considered as a potential subtype of DSM-5 PTSD that applies when Criterion A's implicit assumption that individuals are rationally able to appraise severe threat is violated. In this case, distorted reality trauma would relate to internal or external experiences that are subjectively perceived as involving danger of death, serious injury, or threats to physical integrity. The subtype is not just relevant to samples of people with psychosis but to individuals whose mental state is impaired through other causes, such as highly-medicated intensive care patients who develop PTSD symptoms related to delusions and hallucinations experienced during treatment (Wade et al., 2014) or individuals with dementia (Landqvist Waldö, Gustafson, Passant, & Englund, 2015). Although this proposal is not consistent with the removal of the previous DSM-IV Criterion A_2_ (subjective response) from *DSM-5*, it is more congruent with the lack of a formal stressor criterion in ICD-10 and the proposed *ICD-11* PTSD criteria (Brewin, 2015; Maercker et al., 2013). We recommend that future research investigates the interplay between post-traumatic memory processes and psychotic phenomena. Reminders (e.g., banging noise) of reality-distorted trauma (e.g., past delusional experience of being threatened by the “devil”) might trigger intrusive reliving (PR-PTSD), delusional interpretation (e.g., thinking that memory-based intrusions of a past attack by the “devil” are actually a sign that the devil is attacking again) and/or depressive rumination.

## Conclusions

Although the critical appraisal tool was devised for the current study and may be limited in scope, criteria were based on a systematic review of the literature to identify key factors specific to PR-PTSD research and consensus agreement was reached by clinician researchers with extensive experience in the fields of psychosis and PTSD. Studies did not report all the necessary data to assign quality ratings. It was difficult to disentangle the diverse confounds within studies in order to assess reliability of prevalence rates. Additionally, many studies were of small convenience samples with high refusal rates, and may have suffered from selection bias.

The experience of psychosis has the potential to be traumatic and may lead to a range of maladaptive reactions. Although studies have methodological limitations, there is enough evidence to note that PR-PTSD poses a challenge to DSM-5 because a subset of triggering events will not meet the current Criterion A. We therefore propose that *distorted reality-PTSD* could be considered as a subtype of PTSD occurring when, in the presence of hallucinations or delusions, internal or external events that would not meet *DSM-5* PTSD Criterion A are subjectively appraised as representing a threat of physical harm, serious injury or death. We suggest that, along with a clearer definition, psychometrically robust assessment of PR-PTSD as described in this review is critical to establishing a valid and reliable estimate of its prevalence and ensuring accurate diagnosis and treatment to support people in their recovery from psychosis.

## Footnotes

1 The CAPS establishes if PTSD symptoms persist for one month, but does not evaluate if the specific stressor is still on-going.
